# Magnesium Ethylenediamine Borohydride as Solid-State Electrolyte for Magnesium Batteries

**DOI:** 10.1038/srep46189

**Published:** 2017-04-07

**Authors:** Elsa Roedern, Ruben-Simon Kühnel, Arndt Remhof, Corsin Battaglia

**Affiliations:** 1 Materials for Energy Conversion, Empa - Swiss Federal Laboratories for Materials Science and Technology, 8600 Dübendorf, Switzerland

## Abstract

Solid-state magnesium ion conductors with exceptionally high ionic conductivity at low temperatures, 5 × 10^−8^ Scm^−1^ at 30 °C and 6 × 10^−5^ Scm^−1^ at 70 °C, are prepared by mechanochemical reaction of magnesium borohydride and ethylenediamine. The coordination complexes are crystalline, support cycling in a potential window of 1.2 V, and allow magnesium plating/stripping. While the electrochemical stability, limited by the ethylenediamine ligand, must be improved to reach competitive energy densities, our results demonstrate that partially chelated Mg^2+^ complexes represent a promising platform for the development of an all-solid-state magnesium battery.

Magnesium metal is an attractive anode material for rechargeable batteries, owing to its high volumetric capacity (3833 mAhcm^−3^ compared to 2036 mAhcm^−3^ for Li), negative redox potential of −2.36 V vs. SHE, and abundance. In contrast to metallic Li, Mg can be electroplated dendrite-free and promises consequently high energy density and safe operation^1^,^2^,^3^,^4^. The development of effective Mg electrolytes is a crucial step towards the realization of a competitive reversible Mg battery and significant progress has been made particularly for liquid electrolytes, but safety and stability concerns remain^2^,^5^.

Simple Mg electrolytes using solvents such as propylene carbonate and diethyl carbonate, analogous to typical Li electrolytes, do not allow for reversible electrodeposition of Mg metal, due to the formation of an ion blocking layer on the Mg surface^5^. Most reported Mg electrolytes are therefore based on organometallics, often Grignard reagents, or Mg salts dissolved in ethereal solvents or glymes^1^,^5^,^6^. In particular electrolytes based on magnesium borohydride, Mg(BH_4_)_2_, have received significant attention since 2012, when Mohtadi *et al*. demonstrated the first fully inorganic and halide-free Mg electrolytes enabling reversible Mg plating and stripping^7^,^8^. In an effort to improve the safety, attempts have been made to replace the flammable organic solvents by ionic liquids^9^,^10^,^11^.

By replacing the liquid electrolytes altogether, all-solid-state batteries employing solid-state electrolytes promise to be safer, *e.g*. in terms of heat and mechanical shock resistance. However, designing solid-state electrolyte materials with sufficiently high magnesium ion conductivity represents a major challenge owing to the divalent positive charge carried by the Mg^2+^ ion. Polymer-electrolyte systems, typically based on poly(ethylene oxide) (PEO) and Mg salts were considered as solid-state electrolytes, but the initially investigated salts Mg(SO_3_CF_3_)_2_ or Mg(N(SO_2_CF_3_)_2_)_2_ are known to be incompatible with the Mg metal anode^12^. In inorganic solids, Mg ion conductivity has been reported for few systems, but typically only at high temperatures, *e.g*. phosphate based polycrystalline solid Mg conductors, such as MgZr_4_(PO_4_)_6_ and Mg_1.4_Zr_4_P_6_O_24.4_ + 0.4Zr_2_O(PO_4_)_2_ reach high conductivity above 10^−5^ Scm^−1^ at temperatures above 400 °C^13^,^14^,^15^.

Mg(BH_4_)_2_ derived materials, successfully used as liquid electrolytes thanks to the reductive stability of the BH_4_^−^ anion, are attractive also as solid-state Mg ion conductors. However, the conductivity of pristine Mg(BH_4_)_2_ in the solid state is extremely low (<10^−12^ Scm^−1^ at 30 °C) which has been attributed to its structure, in which the Mg ions are located in firm tetrahedral cages composed of four BH_4_^−^ units^16^. Higher ionic conductivity of 1 × 10^−6^ Scm^−1^ at 150 °C and Mg plating/stripping was demonstrated for Mg(BH_4_)(NH_2_)^17^, in which Mg^2+^ is tetrahedrally coordinated by two BH_4_^−^ and two NH_2_^−^. Reversible Mg plating/stripping was also demonstrated for PEO/Mg(BH_4_)_2_ composites, but no values for the ionic conductivity were reported^18^.

Here we report that high ionic conductivity can be achieved in Mg(BH_4_)_2_ based materials already near room temperature by coordinating Mg^2+^ by a neutral bidentate ethylenediamine ligand (NH_2_CH_2_CH_2_NH_2_, abbreviated as en). Using only one bidentate ligand per metal atom leads to a partially chelated, mixed coordination of Mg^2+^, which renders it mobile. Our approach is compatible with Mg plating/stripping and could be extended to other ligands and Mg salts, widening the portfolio of electrolytes for the development of an all-solid-state Mg battery.

The synthesis of the highly conducting Mg(en)_1_(BH_4_)_2_ proceeds in two steps: First, ball milling of Mg(BH_4_)_2_ with three equivalents of en produces a Mg(en)_3_(BH_4_)_2_ complex. The synthesis, structure, and thermal stability of this compound have been reported recently^19^. In order to have good control over the stoichiometry, ca. 3.2 equivalents of en were used during ball milling and excess en was removed under vacuum at elevated temperatures, see the experimental section in the Supplementary Information for details.

In a second step, the produced Mg(en)_3_(BH_4_)_2_ was ball-milled with Mg(BH_4_)_2_ according to reaction 1 to produce Mg(en)_1_(BH_4_)_2_.


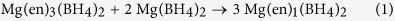


Other ratios of Mg(en)_3_(BH_4_)_2_ and Mg(BH_4_)_2_ were also investigated, however the product with the nominal chemical formula Mg(en)_1_(BH_4_)_2_ showed the highest ionic conductivity.

Powder X-ray diffraction (PXD) shows that the synthesized coordination complex is a polycrystalline solid. Figure 1a compares the diffractograms of Mg(en)_1_(BH_4_)_2_ to those of the reactants Mg(BH_4_)_2_ and Mg(en)_3_(BH_4_)_2_. From comparison of the Bragg reflections, we conclude that Mg(en)_1_(BH_4_)_2_ contains no remaining traces of the reactants Mg(en)_3_(BH_4_)_2_ and Mg(BH_4_)_2_. The first reflection for Mg(en)_1_(BH_4_)_2_ is observed at *q* = 0.49 Å^−1^, indicative of a large unit cell. Indexing of the PXD did not yield a plausible unit cell. Single crystals would facilitate structural analysis, but are not available.

Mg(en)_1_(BH_4_)_2_ is thermally stable up to 75 °C, where a structural phase transition occurs as observed by X-ray diffraction (Figure S1a). The transition is accompanied by an endothermic event observed by differential scanning calorimetry (Figure S1b). A slight weight loss, measured by thermal gravimetry, associated with the decomposition of Mg(en)_1_(BH_4_)_2_, begins above 100 °C (Figure S1b). The thermal stability of Mg(en)_1_(BH_4_)_2_ up to 75 °C is thus comparable to the thermal stability of liquid electrolytes used in standard Li ion batteries.

To shed light onto the local structure and coordination of Mg(en)_1_(BH_4_)_2_, we used Fourier transform infrared (FTIR) and Raman spectroscopy (Fig. 1b,c). We first discuss the coordination of Mg^2+^ in Mg(en)_3_(BH_4_)_2_ for which a crystal structure model has been reported^19^. A conceptual sketch of the local coordination is shown in Fig. 1e. Mg^2+^ is octahedrally coordinated by three chelating bidentate en ligands, resulting in an effectively larger cation [Mg(en)_3_]^2+^ which is charge balanced by the BH_4_^−^ anions.

Going from Mg(en)_3_(BH_4_)_2_ to Mg(en)_1_(BH_4_)_2_, the number of en ligands per Mg^2+^ ion reduces from three to one. Consequently, Mg^2+^ is necessarily forced into a mixed coordination with both en and BH_4_^−^ in the first coordination sphere. In Mg(en)_1_(BH_4_)_2_, two likely possibilities exist for the coordination of Mg^2+^ by the en ligand and BH_4_^−^, as sketched in Fig. 1f and g: (i) A chelating coordination of en as in Mg(en)_3_(BH_4_)_2_, whereby the en ligand is non-bridging in the lower symmetry cis configuration as in Fig. 1f, and the complex could be formulated as [Mg(en)(BH_4_)_2_] or **ii**) a complex in which en acts as a bridging ligand between different Mg^2+^ ions as in Fig. 1g, thus being in the centrosymmetric trans configuration and forming chains, formulated as [-Mg(BH_4_)_2_-en-Mg(BH_4_)_2_-en-].

Evidence favoring the chelating cis configuration comes from FTIR and Raman spectroscopy. The spectra shown in Fig. 1c and d of the starting materials and Mg(en)_1_(BH_4_)_2_ differ significantly. The peak positions are summarized in Table S1 and the descriptions are based on literature assignments of a number of transition metal ethylenediamine complexes studied by vibrational spectroscopy supported by N-deuteration^20^,^21^,^22^,^23^. The FTIR spectrum of Mg(BH_4_)_2_ is characterized mainly by B-H stretching at 2266 cm^−1^, and bending at 1259, 1130 and 1070 cm^−1^. The B-H stretching band splits into several peaks in the FTIR and Raman spectra of Mg(en)_3_(BH_4_)_2_ and Mg(en)_1_(BH_4_)_2_, indicative of the presence of several different types of B-H bonds in the crystal structures, in agreement with four different BH_4_ sites in Mg(en)_3_(BH_4_)_2_. When comparing the spectra of Mg(en)_3_(BH_4_)_2_ and Mg(en)_1_(BH_4_)_2_, the bands assigned to the C-N and C-H backbone of the en ligand in Mg(en)_3_(BH_4_)_2_ and Mg(en)_1_(BH_4_)_2_, remain almost unchanged, namely C-H stretching at 2888 ± 1, 2941 ± 4 and 2963 ± 1 cm^−1^, C-H bending at 1460 ± 2 cm^−1^, CH_2_ twist at 1279 ± 1 cm^−1^ and C-N and C-C stretching at 1094 ± 3 cm^−1^ and 1005 ± 1 cm^−1^, respectively. Additional peaks are present in the spectrum for Mg(en)_1_(BH_4_)_2_ at 3320, 3280 and 3205 cm^−1^ in the N-H stretching region as well as between 1240 and 1120 cm^−1^. A bridging en ligand in trans *C*_*2h*_ configuration results in higher symmetry and thus less peaks in the spectra^21^. This is not observed for the Raman and FTIR spectra of Mg(en)_1_(BH_4_)_2_, where the same peaks are present as in Mg(en)_3_(BH_4_)_2_, evidencing a chelating coordination of en around the Mg ion, as shown in Fig. 1f.

The temperature dependence of the ionic conductivity of Mg(BH_4_)_2_, Mg(en)_3_(BH_4_)_2_ and Mg(en)_1_(BH_4_)_2_ was determined by electrochemical impedance spectroscopy (EIS) in the temperature range between room temperature and 70 °C in symmetric cells with Mg-blocking molybdenum electrodes, Mo/Mg(en)_*x*_(BH_4_)_2_/Mo (Fig. 2a). Typical Nyquist plots are presented in Fig. 2b and c for Mg(en)_1_(BH_4_)_2_ at 30 and 70 °C, respectively and show standard ionic conduction behavior with a single semicircle. The ionic conductivity was extracted from the higher frequency intercept of the extrapolated semicircle with the horizontal axis, and taking into account the geometry of the sample. Ionic conductivities measured as a function of temperature are summarized in Fig. 2a for Mg(BH_4_)_2_, Mg(en)_3_(BH_4_)_2_ and Mg(en)_1_(BH_4_)_2_, together with selected literature data for Mg(BH_4_)(NH_2_), MgZr_4_(PO_4_)_6_ and Mg_1.4_Zr_4_P_6_O_24.4_ + 0.4Zr_2_O(PO_4_)_2_.

The ionic conductivity of Mg(en)_1_(BH_4_)_2_ is 5 × 10^−8^ Scm^−1^ at 30 °C, exceeding that of Mg(BH_4_)_2_ by more than four and that of Mg(en)_3_(BH_4_)_2_ by three orders of magnitude and further increases to above 6 × 10^−5^ Scm^−1^ at 70 °C. This is so far the highest conductivity measured for solid-state Mg conductors in this temperature range. The temperature dependence of the conductivity of Mg(en)_3_(BH_4_)_2_ and Mg(en)_1_(BH_4_)_2_ shows Arrhenius behavior from 30 to 70 °C, with an apparent activation energy of 0.9 and 1.6 eV, respectively. These activation energy values are relatively high but comparable to the other Mg solid-state electrolyte systems reported in Fig. 2a.

Our impedance results show that introducing the chelating en ligand significantly improves the mobility of the Mg^2+^ ion. Of fundamental importance is the reduction of the number of en ligands coordinating the Mg^2+^ cation from three in Mg(en)_3_(BH_4_)_2_ to one in Mg(en)_1_(BH_4_)_2_, leading to an asymmetric mixed coordination of Mg^2+^ by one en and two BH_4_^−^. A related observation was also made for liquid electrolytes, in which Mg(BH_4_)_2_ is solvated by glymes, and where cations which were only partially solvated by the solvent and partially coordinated by the anion were found to be more mobile^18^. However, the findings from liquid chelated systems cannot be translated directly to the solid, since in liquid electrolytes the ion moves with its solvation shell.

Interestingly, impedance measurements on the related halide complexes Mg(en)_1_*X*_2_ (*X* = I, Cl) (not shown here) show much lower ionic conductivity, indicating that the lower symmetry and rotational degrees of freedom of the tetrahedral BH_4_^−^ anions play an important role in the conduction mechanism. To understand this in more detail, the crystal structure of Mg(en)_1_(BH_4_)_2_ will need to be determined.

High ionic conductivity is only one of the requirements for an electrolyte for Mg batteries. To test the stability and to confirm that the observed conductivity stems from the transport of Mg ions, cyclic voltammetry measurements were conducted on an asymmetric Pt/Mg(en)_1_(BH_4_)_2_/Mg cell at 60 °C, as shown in Fig. 3. At −0.2 V vs. the Mg counter electrode, we observe the onset of cathodic current corresponding to Mg plating onto the Pt working electrode. The anodic current during the reverse sweep between −0.2 V and 0.5 V is attributed to Mg stripping, strongly indicating that Mg conduction indeed takes place.

For voltages >1.2 V, we observe irreversible oxidation. The interface layer formed during this irreversible oxidation is able to conduct Mg ions, since plating/stripping is also observed in the following cycles. Indeed, the plating/stripping currents increase during the first seven cycles (see Fig. 3 cycles and inset), suggesting that an initialization period improves the interfacial contact between electrodes and electrolyte

To provide direct evidence for Mg^2+^ ion conduction through the electrolyte, we assembled a Cu/Mg(en)_1_(BH_4_)_2_/Mg cell and deposited Mg on the Cu electrode at 60 °C. The cell was subsequently disassembled and the surface of the Cu electrode examined by scanning electron microscopy (SEM) and energy dispersive X-ray (EDX) analysis. The SEM image of the Cu electrode in Figure S3 shows randomly distributed patches, identified as Mg by EDX (also shown in Figure S3), which confirm Mg plating. No such patches were observed for an identically build reference cell to which no plating current was applied (Figure S4). The patchy deposition of Mg provides additional evidence that electrolyte and Cu electrode are not in contact over the full area, but that the contact area increases upon plating, as the plated Mg fills the gaps between electrolyte and electrode. Also for liquid electrolyte systems it has been observed that newly formed interfaces between Mg metal and electrolyte improve the performance^24^.

In this work, we demonstrate that partially chelated ethylenediamine magnesium borohydride complexes are promising new solid-state Mg conductors with high conductivity of up to 5 × 10^−8^ Scm^−1^ at 30 °C and 6 × 10^−5^ Scm^−1^ at 70 °C, tackling the challenge of mobility of the divalent Mg^2+^ ion in the solid state. The compounds are easily synthesized by ball milling, no sintering or other energy expensive processes were necessary. While Mg plating/stripping proved feasible, more studies are necessary to understand the mechanism of ionic conductivity on the atomic level in these systems and the interface dynamics. Our approach using partially chelated Mg complexes with a complex anion as solid-state Mg electrolytes can be extended to other ligands and anions, allowing for the tuning of the electrochemical stability. This could open the doors for the development of all solid-state high energy density Mg batteries, conceivably a game-changer in battery technology.

## Methods

### Sample Synthesis

The synthesis of Mg(en)_3_(BH_4_)_2_ was carried out according to a modified literature procedure^19^. Mg(BH_4_)_2_ was ball-milled with approximately 3.2 equivalents ethylenediamine (en) in a Spex 8000 shaker mill under argon atmosphere for 3 × 10 min, with 5 min break in between, using a balls-to-sample mass ratio of 10 to 1. An excess of en with respect to the molar ratio for Mg(en)_3_(BH_4_)_2_ was used to ensure a complete reaction. The excess en was removed from Mg(en)_3_(BH_4_)_2_ under dynamic vacuum at 120 °C (1 h). For the synthesis of Mg(en)_1_(BH_4_)_2_, the produced Mg(en)_3_(BH_4_)_2_ was ball-milled with additional Mg(BH_4_)_2_ in the correct stoichiometric ratio, using the same ball milling procedure.

The chemical formula of Mg(en)_1_(BH_4_)_2_ and the stoichiometric reaction according to (1) is corroborated by temperature dependent PXD between 30 and 70 °C, which shows the simultaneous decrease of the intensity of reflections of both reactants, while the new compound is formed (see Figure S2). In the same temperature range, thermogravimetric analysis of the reaction mixture records no weight loss.

All preparations and manipulations of samples were performed in a glove box with a circulation purifier (argon atmosphere, <1 ppm of O_2_ and H_2_O). The chemicals magnesium borohydride Mg(BH_4_)_2_ (Aldrich, 95%) and ethylenediamine (Aldrich, 99.5%) were used as received.

### Structural Characterization

Powder X-ray diffraction (PXD) measurements were performed on a Bruker D8 Advance diffractometer equipped with a Goebel mirror selecting Cu Kα radiation (λ = 1.5418 Å) and a linear detector system (Vantec). Samples were filled and sealed into glass capillaries under argon atmosphere. Simultaneous differential scanning calorimetry and thermogravimetry measurements were performed using a Netzsch STA 449 F3 Jupiter with Al sample pans at Δ*T*/Δ*t* = 5 Kmin^−1^ between 30 and 400 °C under He flow at 80 ml min^−1^.

FTIR spectra were obtained between 900 and 4000 cm^−1^ with a Bruker Vektor 20 spectrophotometer and a Golden Gate ATR cell in atmospheric conditions, causing short air exposure during the measurements. Raman measurements were performed on samples packed in glass capillaries between 100 and 4000 cm^−1^, using a Reninshaw Raman system equipped with a HeNe Laser, λ = 633 nm.

### Electrochemical Characterization

Electrolyte pellets were pressed using a pressing mold with an inner diameter of 12 mm. The material was weighed and loaded into the mold and pressed using a knuckle joint press inside the glovebox. The thickness of the resulting pellet was determined with a caliper (between 0.5 and 0.8 mm). Electrochemical measurements were performed in 2-electrode stainless steel cells, which were assembled inside an argon filled glovebox. A spring was used to maintain mechanical contact between electrodes and electrolyte.

Electrochemical impedance spectroscopy (EIS) was measured using a Zahner IM6ex electrochemical workstation in the frequency range 3 × 10^6^ − 0.1 Hz. Polished molybdenum blocks were used as Mg blocking electrodes. Impedance spectra were obtained at 30, 40, 50, 60 and 70 °C, after thermal equilibration for 2 h at each temperature.

Cyclic voltammetry (CV) measurements were performed at 60 °C after thermal equilibration for 4 h with a Biologic VMP3 electrochemical workstation. An asymmetric cell was prepared with Mg foil (99.95% Mg, 11 mm diameter, 0.05 mm thickness, GalliumSource) as counter electrode and Pt as working electrode. The OCV stabilized at 0.66 V and cyclic voltammetry was carried out between −0.5 and 1.7 V with a scan rate of 10 mVs^−1^. A similar cell, but with a Cu working electrode was built. Mg was plated at a current of 3 nA for ca. 200 h at overpotentials below −0.1 V at 60 °C. SEM images were taken with a FEI NanoSEM 230 at 25 kV. EDX analysis was performed with an Oxford X-Max SDD EDX system.

## Additional Information

**How to cite this article**: Roedern, E. *et al*. Magnesium Ethylenediamine Borohydride as Solid-State Electrolyte for Magnesium Batteries. *Sci. Rep.* **7**, 46189; doi: 10.1038/srep46189 (2017).

**Publisher's note:** Springer Nature remains neutral with regard to jurisdictional claims in published maps and institutional affiliations.

## Supplementary Material

Supplementary Information

Supplementary Dataset 1

Supplementary Dataset 2

Supplementary Dataset 3

Supplementary Dataset 4

Supplementary Dataset 5

Supplementary Dataset 6

## Figures and Tables

**Figure 1:**
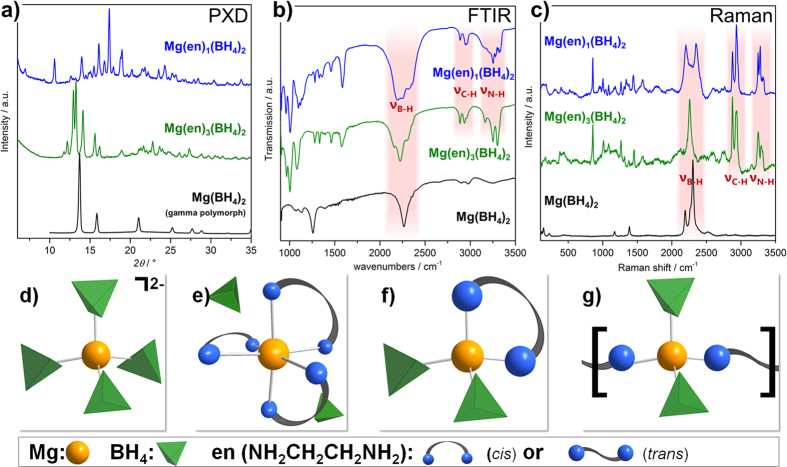
(**a**) Powder X-ray diffractograms, (**b**) FTIR and (**c**) Raman spectra of Mg(BH_4_)_2_, Mg(en)_3_(BH_4_)_2_, and Mg(en)_1_(BH_4_)_2_ along with a conceptual sketch of the local coordination of Mg^2+^ in (**d**) Mg(BH_4_)_2_, (**e**) Mg(en)_3_(BH_4_)_2_, and two likely possibilities in Mg(en)_1_(BH_4_)_2_ (**f**,**g**).

**Figure 2:**
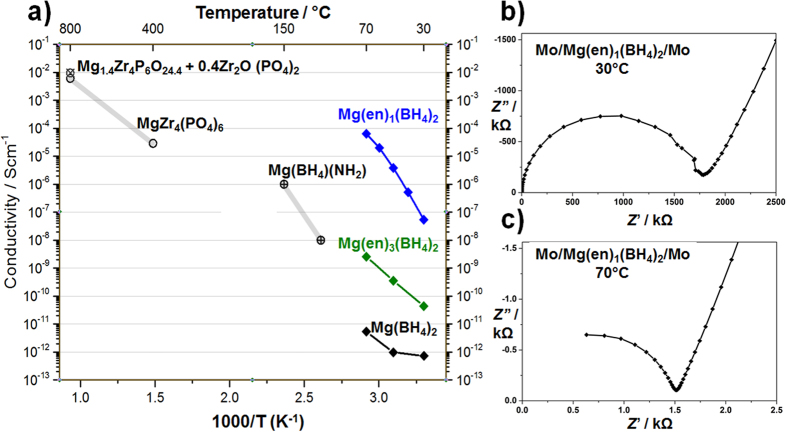
(**a**) Temperature dependence of ionic conductivity of Mg(BH_4_)_2_, Mg(en)_3_(BH_4_)_2_ and Mg(en)_1_(BH_4_)_2_, together with literature data for MgZr_4_(PO_4_)_6_^13^, Mg_1.4_Zr_4_P_6_O_24.4_ + 0.4Zr_2_O(PO_4_)_2_^14^,^15^ and Mg(BH_4_)(NH_2_)^17^. (**b**,**c**) Nyquist plots of Mo/Mg(en)_1_(BH_4_)_2_/Mo at 30 and 70 °C, respectively.

**Figure 3:**
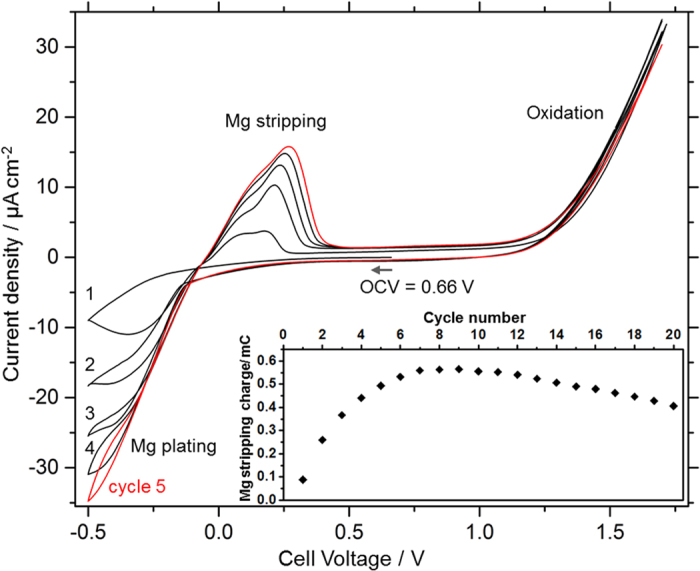
Cyclic voltammogram of a Pt/Mg(en)_1_(BH_4_)_2_/Mg cell at 60 °C at a scan rate of 10 mVs^−1^. Inset: Mg stripping charge (=area of the Mg stripping peak) vs. cycle number.
