# Supplementary material for: Magnesium Ethylenediamine Borohydride as Solid-State Electrolyte for Magnesium Batteries
Source: Sci Rep. 2017 Apr 7;7:46189. doi: 10.1038/srep46189 (PMC5384009; doi:10.1038/srep46189)
Supplement: Supplementary Information [file srep46189-s1.pdf]

Magnesium Ethylenediamine Borohydride as Solid-State Electrolyte for Magnesium Batteries

Elsa Roedern, Ruben-Simon Kühnel, Arndt Remhof and Corsin Battaglia*

Supporting Information

Table of Content

- Supporting Figures and Tables
  - Figure S1. a) *In situ* PXD of $Mg(en)_1(BH_4)_2$ during heating from 30 to 100 °C. b) Differential scanning calorimetry (DSC) and c) Thermogravimetric analysis (TGA) of $Mg(en)_3(BH_4)_2$ and $Mg(en)_1(BH_4)_2$.
  - Figure S2. *In situ* PXD of $Mg(en)_3(BH_4)_2$ + 2 $Mg(BH_4)_2$ during heating from 30 to 85 °C.
  - Table S1. Infrared (FTIR) and Raman peaks of $Mg(BH_4)_2$, $Mg(en)_3(BH_4)_2$ and $Mg(en)_1(BH_4)_2$
  - Figure S3. SEM image and elemental EDX maps of the Cu electrode after Mg plating in a Cu/$Mg(en)_1(BH_4)_2$/Mg cell at 60 °C.
  - Figure S4. SEM image and elemental EDX maps of Cu electrode in the reference cell Cu/$Mg(en)_1(BH_4)_2$/Mg, kept at 60 °C, but to which no plating current was applied.
- References

Supporting Figures and Tables

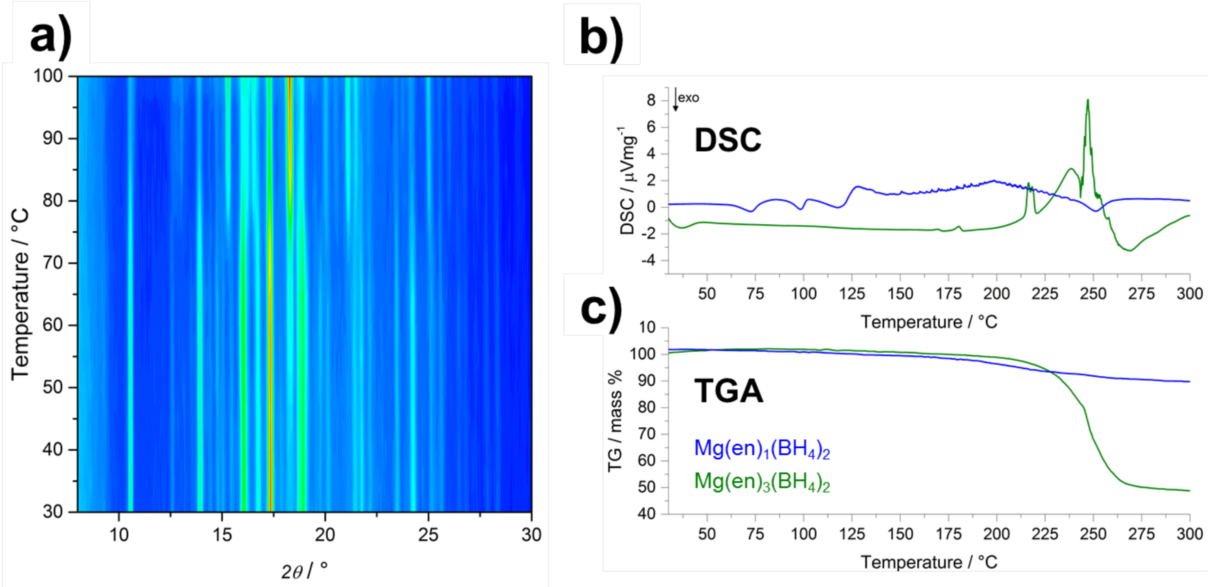

Figure S1. a) *In situ* PXD of Mg(en)$_1$(BH$_4$)$_2$ during heating from 30 to 100 °C. b) Differential scanning calorimetry (DSC) and c) Thermogravimetric analysis (TGA) of Mg(en)$_3$(BH$_4$)$_2$ and Mg(en)$_1$(BH$_4$)$_2$, 30 - 300 °C, $\Delta T/\Delta t$ = 5 °C/min, He flow.

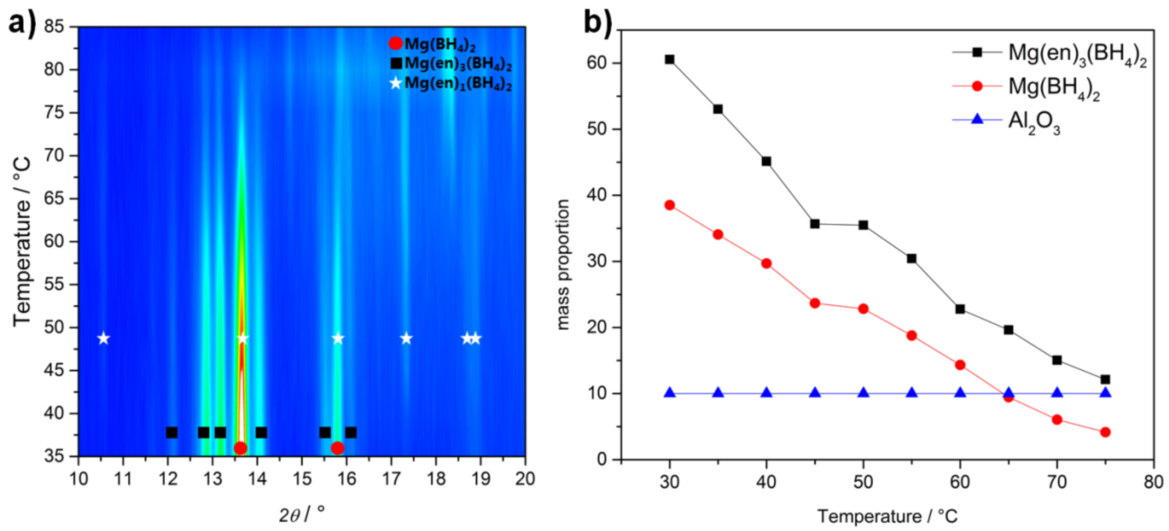

Figure S2. a) *In situ* PXD of Mg(en)$_3$(BH$_4$)$_2$ + 2 Mg(BH$_4$)$_2$ during heating from 30 to 85 °C, b) mass proportion of Mg(en)$_3$(BH$_4$)$_2$, Mg(BH$_4$)$_2$ and Al$_2$O$_3$ (internal standard, normalized to 10 wt%), values obtained by Rietveld refinement.

Table S1. Experimentally observed Infrared (FTIR) and Raman peaks of $Mg(BH_4)_2$, $Mg(en)_3(BH_4)_2$ and $Mg(en)_1(BH_4)_2$, approx. description based on literature references[2–5].

$Mg(BH_4)_2$				$Mg(en)_3(BH_4)_2$				$Mg(en)_1(BH_4)_2$				approx.
FTIR		Raman		FTIR		Raman		FTIR		Raman		Description
...		...		...		...		3332	m	3320	m,b	N-H stretching
...		...		...		...		3319	m	...		N-H stretching
...		...		3301	s	3294	m,b	3303	m	...		N-H stretching
...		...		...		...		3280	m	3279	s	N-H stretching
...		...		3251	s	3250	s	3253	s	3248	s	N-H stretching
...		...		3166	m	3159	w,b	3166	m,sh	3160	w,b	N-H stretching
...		...		2962	m,sh	...		2964	m	...		C-H stretching
...		...		2937	m	2940	s,b	2945	m	2933	s,b	C-H stretching
...		...		2887	m	2876	s	2889	m	2877	s	C-H stretching
...		...		...		...		2322	s,b	2350	s	B-H stretching
...		2305	s	2300	s/sh	...		...		...		B-H stretching
2266	S	2267	m,sh	...		2256	s	2254	s,sh	2256	m,b	B-H stretching
...		...		2226	s	...		...		...		B-H stretching
...		2193	m	...		...		2198	s,b	2204	s	B-H stretching
...		...		2158	s,sh	...		...		...		B-H stretching
...		...		1602	w,sh	...		...		...		N-H bending
...		...		1573	m	1574	w,b	1585	s	1577	w,b	N-H bending
...		...		1461	m	1454	m	1458	m	1443	m	C-H bending
...		1387	m	...		1378	w,b	...		1380	w,b	B-H bending
...		...		...		...		1359	m,b	...		
...		...		...		...		...		1340	m,b	
...		...		1332	m	...		1328	m	...		NH2 wagging
...		...		...		1315	w	...		1310	w,b	
...		...		...		...		...		...		
...		...		1278	m	1262	m	1280	m	1265	m	CH2 gamma
1259	S	...		...		...		1240	w,b	...		B-H bending
...		...		...		...		...		1182	m,b	
...		1171	m,b	...		...		1160	w,sh	1162	w,b	B-H bending
1130	m,b	...		...		...		1143	m,sh	...		B-H bending
...		...		...		...		1120	m, sh	...		
...		...		1090	b,sh	1084	m,b	1097	m,b	1084	m	C-N stretching
1070	w,b	...		1080	b,sh	...		1070	w, sh	...		B-H bending
...		...		1004	s	1012	m,b	1006	s	1003	m	C-C stretching
...		...		968	s	960	w,b	960	s	971	w	NH2 wagging
...		...		910	m	...		...		951	w	
...		...		...		...		908	m	...		
...		...		...		856	s	...		856	s	

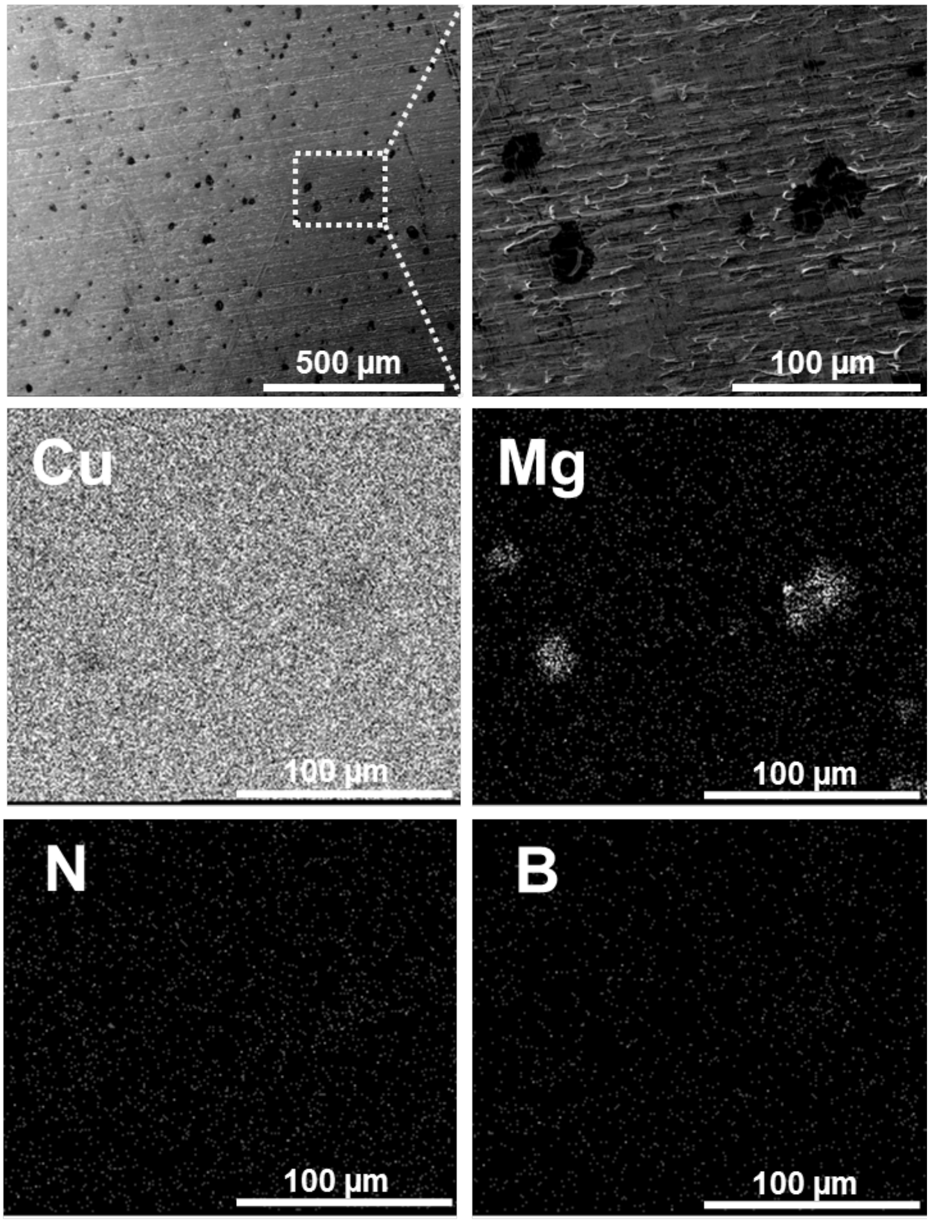

Figure S3. SEM image and elemental EDX maps of the Cu electrode after Mg plating in a $Cu/Mg(en)_1(BH_4)_2/Mg$ cell at 60 °C. The patches are identified by EDX as Mg without N and B.

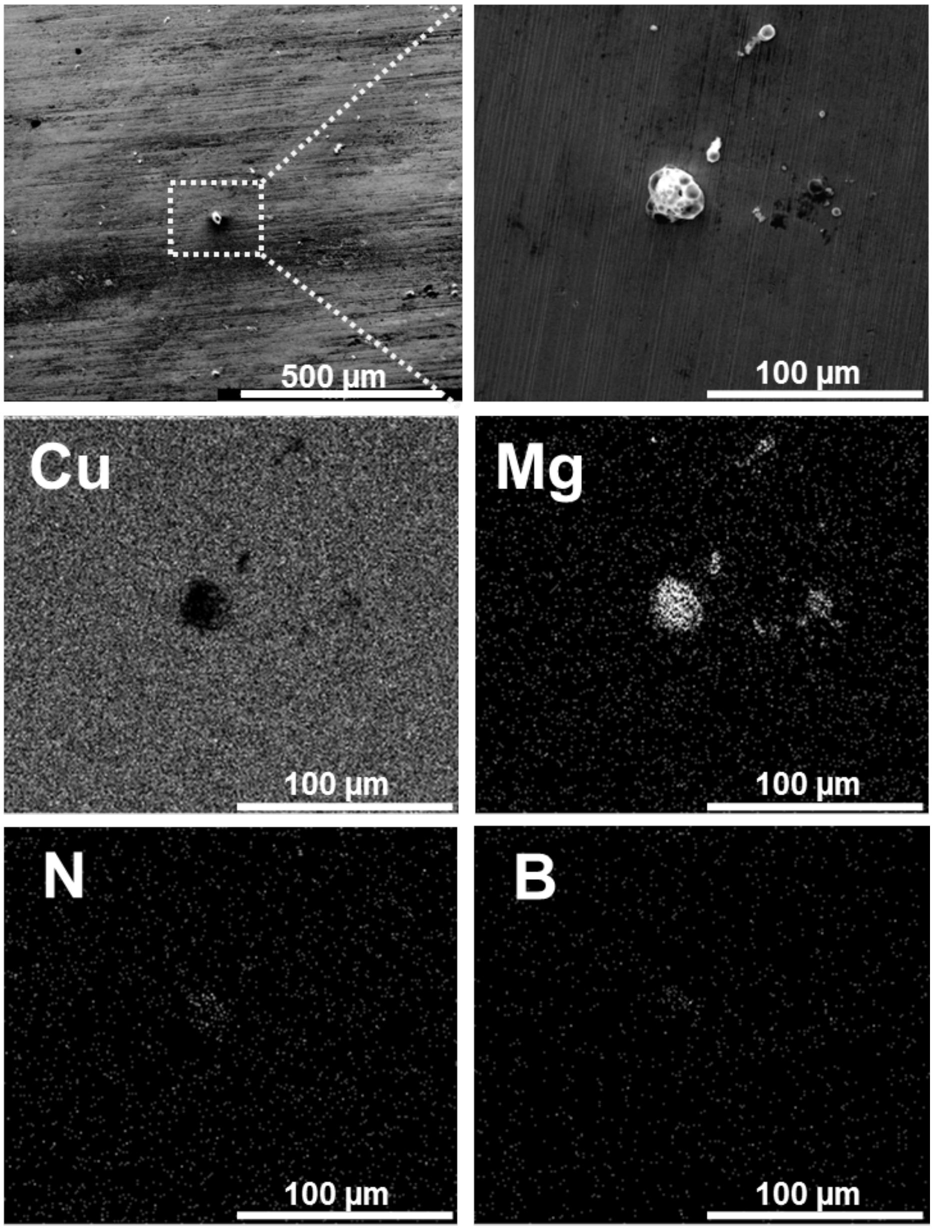

Figure S4. SEM image and elemental EDX maps of Cu electrode in the reference cell $Cu/Mg(en)_1(BH_4)_2/Mg$, kept at 60 °C, but to which no plating current was applied. A few particles can be found on the Cu electrode surface, which are identified by EDX as electrolyte particles with Mg, N and B.

References

[1] J. Chen, Y. S. Chua, H. Wu, Z. Xiong, T. He, W. Zhou, X. Ju, M. Yang, G. Wu, P. Chen, *Int. J. Hydrogen Energy* **2015**, *40*, 412–419.

[2] M. G. Giorgini, M. R. Pelletti, G. Paliani, R. S. Cataliotti, *J. Raman Spectrosc.* **1983**, *14*, 16–21.

[3] T. Iwamoto, D. F. Shriver, *Inorg. Chem.* **1971**, *10*, 2428–2432.

[4] A. D. Allen, C. V Senoff, *Can. J. Chem.* **1965**, *43*, 888–895.

[5] A. M. A. Bennett, G. A. Foulds, D. A. Thornton, G. M. Watkins, *Spectrochim. Acta A* **1990**, *46*, 13–22.
